# *Re A (A Child)* and the United Kingdom Code of Practice for the Diagnosis and Confirmation of Death: Should a Secular Construct of Death Override Religious Values in a Pluralistic Society?

**DOI:** 10.1007/s10730-016-9307-y

**Published:** 2016-08-04

**Authors:** Kartina A. Choong, Mohamed Y. Rady

**Affiliations:** 1 Lancashire Law School, University of Central Lancashire, Corporation Street, Preston, PR1 2HE UK; 2 Department of Critical Care Medicine, Mayo Clinic Hospital, 5777 East Mayo Boulevard, Phoenix, AZ 85054 USA

**Keywords:** Brainstem death, Code of practice, Determination of death, Disorders of consciousness, Law, Life-support treatment, Neuroscience, Religious ethics

## Abstract

The determination of death by neurological criteria remains controversial scientifically, culturally, and legally, worldwide. In the United Kingdom, although the determination of death by neurological criteria is not legally codified, the Code of Practice of the Academy of Medical Royal Colleges is customarily used for neurological (brainstem) death determination and treatment withdrawal. Unlike some states in the US, however, there are no provisions under the law requiring accommodation of and respect for residents' religious rights and commitments when secular conceptions of death based on medical codes and practices conflict with a traditional concept well-grounded in religious and cultural values and practices. In this article, we analyse the medical, ethical, and legal issues that were generated by the recent judgement of the High Court of England and Wales in *Re: A (A Child)* [2015] EWHC 443 (Fam). Mechanical ventilation was withdrawn in this case despite parental religious objection to a determination of death based on the code of practice. We outline contemporary evidence that has refuted the reliability of tests of brainstem function to ascertain the two conjunctive clinical criteria for the determination of death that are stipulated in the code of practice: irreversible loss of capacity for consciousness and somatic integration of bodily biological functions. We argue that: (1) the tests of brainstem function were not properly undertaken in this case; (2) the two conjunctive clinical criteria set forth in the code of practice cannot be reliably confirmed by these tests in any event; and (3) absent authentication of the clinical criteria of death, the code of practice (in fact, although implicitly rather than explicitly) wrongly invokes a secular definition of death based on the loss of personhood. Consequently, the moral obligation of a pluralistic society to honor and respect diverse religious convictions to the greatest extent possible is being violated. *Re A (A Child)* is contrasted with the US case of Jahi McMath in which the court accommodated parental religious objection to the determination of neurological death codified in the Uniform Determination of Death Act. We conclude that the legal system in the United Kingdom should not favour a secular definition of death over a definition of death that is respectful of religious values about the inviolability and sanctity of life. We recommend the legal recognition of religious accommodation in death determination to facilitate cultural sensitivity and compassionate care to patients and families in a pluralistic society.

**Electronic supplementary material:**

The online version of this article (doi:10.1007/s10730-016-9307-y) contains supplementary material, which is available to authorized users.

## Introduction

Brain death or the determination of death by neurological criteria remains controversial scientifically, culturally, and legally, worldwide (Bernat 2015). There is no uniform neurological standard in death determination because of the variability in practices and perceptions of the global medical community about brain death (Wahlster et al. 2015). The neurological determination of death varies among countries with regard to the use of whole brain criteria or brainstem criteria, and the mandatory performance of confirmatory tests (Ding et al. 2015; Citerio and Murphy 2015; Chua et al. 2015; Varelas 2016; Cameron et al. 2016). Confirmatory tests include neuroimaging of cerebral blood flow and electrophysiological studies of the cerebral hemispheres and the brainstem.

In the United Kingdom (UK), neurological death is determined by performing a particular set of tests for brainstem function (Table 1). These are in accordance with, and outlined in, the Academy of Medical Royal Colleges’ “Code of Practice for the Diagnosis and Confirmation of Death” which was first issued in 1976 and subsequently updated (The Academy of Medical Royal Colleges 2008). Although there is, to date, no legislation which confirms the legitimacy of the code of practice, courts have consistently endorsed it in the determination of legal death (*R v. Malcherek and R v. Steel* 1981; *Re A (A Minor)* 1992). The judicial system has always assumed that properly undertaken tests of brainstem function can reliably confirm the two conjunctive clinical criteria of death (clinical death) set forth in the code of practice: irreversible loss of capacity for consciousness and somatic integration of bodily biological functions. *Ipso facto*, all life-support treatment, including mechanical ventilation, can legally be withdrawn once brainstem death is determined.

**Table 1:** The criteria for diagnosis of brainstem death

Eligibility criteria	Reversible confounding factors^a^	Tests of brainstem function (reflexes) in brainstem death
Unresponsiveness to external stimuli (coma) Dependency on mechanical ventilation Known cause of unresponsiveness or presence of structural brain injury Period of observation	Residual pharmacological effects e.g., sedatives, opioids, general anesthesia, neuro-muscular blockers Thermoregulatory disturbances Endocrine abnormalities Metabolic derangements	No pupillary response to light No corneal reflex No vestibule-ocular reflex (Caloric test) No Ocular-cephalic reflex (Doll’s eye) No motor response to pain—in the trigeminal nerve distribution No gag reflex in response to suction through endotracheal tube or tracheostomy Apnea persists despite a rise in PaCO_2_ to greater than 50 mmHg (6.6 kPa) against a background of a normal PaO_2_

Adapted from the source: “A code of practice for the diagnosis and confirmation of death. A report of the Academy of Medical Royal Colleges” (The Academy of Medical Royal Colleges 2008)

^a^Confounding factors are clinical conditions that can reversibly suppress brainstem function and result in an erroneous diagnosis of brainstem death. Failure to recognize the presence of confounding factors and a false-positive brainstem death determination can result in premature termination of life-support treatment

This approach was recently challenged in *Re: A (A Child)* [2015] EWHC 443 (Fam). In this heart-rending and tragic case, the Muslim parents of a brainstem dead child opposed the switching off of his mechanical ventilator on grounds of religious objection to a determination of death based on the code of practice. The High Court of England and Wales dismissed their challenge to death determination as coming from distraught parents who were unable to accept the death of their child. It went on to permit the immediate non-consensual withdrawal of the child’s life-support treatment including mechanical ventilation. In choosing not to engage with the arguments the parents put forward in furtherance of their religious observance of the Islamic faith, the court seemed to have given short shrift to their plea for dissension from the secular and scientific code.

We argue in this article that this is potentially a problematic stance. From a medical perspective, we highlight two challenging questions in *Child A*’s case: one, was the code of practice applied at the appropriate time after acute hypoxic–ischemic brain injury; and two, is the code of practice used in the determination of death scientifically valid and thus, arguably, authoritative? In answering both these questions in the negative, we argue that the judiciary should have exercised more caution in endorsing the application of the code in the determination of death. Further and more importantly, it should have made some effort towards accommodating and respecting residents' religious rights and commitments when secular conceptions of death based on medical codes and practices conflict with a traditional concept well-grounded in religious and cultural values and practices.

We begin the discussion by analysing the facts of the case and the judgment delivered by Mr. Justice Hayden (see the Supplementary File). We then provide a critique of the approach taken in the case from medical and ethical perspectives. For comparative analysis, we refer to the United States’ (US) practices. We conclude with recommendations that can enhance religious and cultural sensitivity in life-ending medical decision-making in a pluralistic society like the UK.

## *Re A (A Child)*: A Missed Opportunity?

Child A, who was 19 months old, choked on a tiny piece of fruit on the 6th of February 2015 and was immediately taken to the Central Manchester University Hospital (*Re: A (A Child)* 2015, para 3). On arrival at the hospital, he was in severe respiratory distress and soon went into cardiac arrest at 15.11 h. In addition to receiving CPR and being intubated, he was operated on to remove the foreign object. He was successfully resuscitated from cardiac arrest at 15.31 h and transferred to pediatric intensive care where he continued to be heavily sedated and mechanically-ventilated. A neuro-protective regime was initiated (*Re: A (A Child)* 2015, para 5). Brainstem tests were performed at 10.10 a.m. and 5.30 p.m. on the 10th of February 2015 and indicated death by brainstem criteria (*Re: A (A Child)* 2015, para 1). Medical staff concluded that mechanical ventilation should be discontinued because the child was clinically and legally dead in line with the code of practice and established case law.

The coroner, who was also informed of the development, wrote to the hospital Trust’s clinical director when the mechanical ventilator was not discontinued 2 days after brainstem death determination, stating that:

Technically, I have assumed jurisdiction over the body. It seems wholly inappropriate for a deceased body to be intubated and ventilated when this is futile and, to my mind, unethical. Accordingly, I must ask you to cease this and extubate him so that his body can be moved to the mortuary from which it can be released to his parents (*Re: A (A Child)* 2015, para 19).

The delay in withdrawing mechanical ventilation was precipitated by Child A’s parents’ refusal to accept brainstem death as synonymous with death in the Islamic faith. The signs that distinguish between life and death are well-described in the Islamic scriptures (Rady and Verheijde 2016c). From a religious viewpoint, death is demarcated by the soul’s departure from the body. The soul’s presence is associated with the continuation of a beating heart and the perpetuation of breathing, even if aided by artificial ventilation (Bedir and Aksoy 2011, pp. 290–291), as was the case with Child A; consequently, this person is still considered alive.

Because of the disagreement between the parents and the medical staff and coroner on death determination, the hospital sought a declaration from the High Court to discontinue life-support treatment, including mechanical ventilation. In granting this declaration, Mr. Justice Hayden re-confirmed the stance taken in earlier case law: that a person on mechanical ventilation is legally dead when brainstem death has been confirmed. Child A was declared to have died at 10.10 a.m. on the 10th of February 2015 (*Re: A (A Child)* 2015, para 1). Although Mr. Justice Hayden acknowledged that “in a multi-cultural society there has to be recognition that people, particularly those with strong religious beliefs, may differ with medical professional as to when death occurs” (*Re: A (A Child)* 2015, para 24), it was remarked that Child A’s parents’ objection was rooted not just in their Muslim beliefs, but also in their “basic parental instinct” (*Re: A (A Child)* 2015, para 10). The parents had, he observed, “simply been unable to contemplate turning off ventilator support,” and desperately therefore “[clung] on to any sign that may undermine these catastrophic medical conclusions” (*Re: A (A Child)* 2015, para 17) and “cleave[d] to what thread of life [they perceived their] son still to have” (*Re: A (A Child)* 2015, para 7).

The High Court also refused to consider the parents’ alternative request for the continuation of mechanical ventilation until the child is repatriated to Saudi Arabia since they were Saudi citizens (the child’s father was studying for a PhD in the UK). By allowing the continuation of mechanical ventilation, the parents could have had enough time to arrange the transfer of Child A to a medical facility in Saudi Arabia, where medical treatment would be continued (*Re: A (A Child)* 2015, para 18). Instead, Mr. Justice Hayden authorized the immediate discontinuation of mechanical ventilation in what he had already considered to be a corpse on a ventilator (*Re: A (A Child)* 2015, para 14).

Nevertheless, a small concession was made when he emphasized that where discordant views arise in the future between the medical staff and the patient’s family about continuation of assisted mechanical ventilation, the dispute should be determined in the High Court rather than under coronial powers (*Re: A (A Child)* 2015, para 27). Minimally, this gives the patient’s ambivalent “dead or alive” status the benefit of the doubt.

Given the unique facts above, this case seems to be the first brought to the British courts where the objection to the prevailing definition of death was registered on the grounds of religion in general and the Islamic faith in particular. By refusing to engage with the challenges mounted against the universal applicability of the code, the ruling may have represented a missed opportunity to develop an ethically-robust and religiously-sensitive approach to the determination of death in the UK. The ensuing discussion will explore the medical and ethical challenges surrounding this case.

## A Medico-Ethical Critique

### The Medical Perspective

Reflecting on the medical facts of *Re A (A Child)*, there are two issues that warrant close examination. First, was the medical evidence presented to Mr. Justice Hayden sufficient to ensure that the code of practice was applied in *Child A* at the appropriate time after acute hypoxic–ischemic brain injury, and therefore to warrant a finding of neurological (brainstem) death under the code of practice? The medical evidence provided to the Court consisted of neurological death determination by brainstem criteria as stipulated in the code of practice, which included the 1991 report of the British Pediatric Association on the diagnosis of brainstem death in infants and children. Second, is the code of practice, even when applied appropriately, valid proof of “clinical death”?

Neurological (brainstem) death was determined by “simple bedside tests” of brainstem function (Table 1) (*Re: A (A Child)* 2015, para 9). As presented to Mr. Justice Hayden by Dr. Playfor (the consultant pediatric intensivist responsible for Child A), “[t]he key point…is that no patient has ever regained consciousness or awareness following brain stem death” (*Re: A (A Child)* 2015, para 11). In addressing the first question (was the medical evidence sufficient to discontinue life-support treatment, at that particular time, after hypoxic–ischemic brain injury?) it should be noted that Child A was started on a treatment plan with a “neuro-protective regime” and was also “heavily sedated” (*Re: A* *(A Child)* 2015, para 5). Brainstem tests were performed at 91 h and repeated again at 98 h after resuscitation from cardiac arrest. Reversible suppression of brainstem reflexes can persist longer than 98 h after neuro-protective therapy has been initiated and influence the accuracy of neurological death determination (Webb and Samuels 2011). Recovery of ceased neurological functions can be delayed as long as 278 h after discontinuing sedative drugs and neuro-protective therapy (targeted temperature management) (Paul et al. 2016). Therefore, a minimum waiting time of no less than 120 h has been recommended before determining the irreversibility of hypoxic–ischemic brain injury (Ponz et al. 2016). If *reversible* suppression of brainstem reflexes is mistaken as *irreversible* cessation of brainstem function, neurological death is determined incorrectly (Joffe et al. 2009; Webb and Samuels 2011). Furthermore, the timeline of recovery of higher brain functions and consciousness (awakening) after neuro-protective therapy can be delayed up to 2 weeks following hypoxic–ischemic brain injury (Gold et al. 2014).

The code of practice does not require performing confirmatory tests (absence of cerebral blood flow, electric silence on electroencephalography, and/or absence of evoked potentials) in the determination of neurological death except under exceptional conditions, such as when the clinical examination of brainstem function is not possible or deemed unreliable because of confounding factors (The Academy of Medical Royal Colleges 2008). Confounding factors (Table 1) are clinical conditions that can potentially suppress brainstem reflexes, but are reversible. Therefore, brainstem function could recover with time. Not all confounding factors are accurately recognized in clinical practice. The most common confounding factors are persistent pharmacological effects of sedatives, opioids, anesthetic drugs, neuromuscular-blocking drugs, or metabolic derangements (Cameron et al. 2016). Pharmacological effects of heavy sedation as well as thermoregulatory and metabolic derangements were potential confounding factors present in Child A (*Re: A (A Child)* 2015, para 5 and para 16). When Child A’s father noticed the presence of “twitching and retraction of Child A’s legs”, the medical staff dismissed these movements as “spinal, not cerebral reactions” and proceeded with brainstem death determination (*Re: A (A Child)* 2015, para 17). The code of practice has broadly assumed that limb movements are “independent of the brain and controlled through the spinal cord” (The Academy of Medical Royal Colleges 2008, p. 11). However, movements in lower extremities were also noted in cases of incorrect brain death determination (Morales 2008). These movements could be mediated by the brainstem and do not necessarily originate from the spinal cord alone and, thus, should have stopped clinicians from proceeding with brainstem tests to determine neurological death (Joffe and Anton 2007). As a note of caution, at autopsy, the brainstem was reported as normal or minimally ischemic in about 60 % of patients who were determined brain dead by clinical examination only (Wijdicks and Pfeifer 2008).

Child A was determined brainstem dead after repeating the required tests at a 7-h interval. Failure to perform at least two confirmatory tests and to repeat both the clinical examination and confirmatory tests at a minimum of 12 h-interval has been reported to increase the likelihood of brain-death diagnosis by 370 % (Ding et al. 2015). Failure to comply with these safeguards can increase the diagnostic error rate and the rate of false positive determination of neurological death. Reliance on clinical examination alone can also result in an erroneous determination of neurological death if medical staff fail to recognize the presence of potential confounding factors (Joffe et al. 2010). Such instances have been reported in several cases in which patients regained some of the ceased functions of the brainstem or made a complete neurological recovery after being determined dead by neurological criteria (Morales 2008; Joffe et al. 2009; Marik and Varon 2010; Roberts et al. 2010; Webb and Samuels 2011; Sullivan et al. 2012). Incorrect determination of brainstem death therefore has a lethal consequence because it results in the premature withdrawal of life-support treatment.

Magnetic resonance imaging (MRI) was obtained 24 h after the acute hypoxic–ischemic injury of Child A’s brain. The MRI predictably “revealed extensive severe ischemic changes involving the grey matter of Child A’s brain [and] that the brain injury was so extensive that it was something from which it was impossible for Child A to survive” (*Re: A (A Child)* 2015, para 6). However, the MRI in these cases produces static images of the higher brain structures including the cerebral cortex. It does not measure either the retained activity or function of cerebral neurons. Is it then reasonable to raise the question whether the medical evidence was sufficient to conclude that Child A cannot recover higher brain functions? How reliable was the presence of extensive ischemic changes of the cerebral cortex on a brain MRI in predicting the potential for neurological recovery? Contemporary medical evidence has suggested that neuroimaging cannot reliably predict the neurological outcome in cardiac arrest survivors with hypoxic–ischemic brain injury (Hahn et al. 2014). This is most relevant in infants and children. The developing infant brain has a greater plasticity compared to an adult brain and is more likely to achieve better neurological recovery after extensive ischemic injury on neuroimaging.

Regarding the second question (Is the code of practice, when applied appropriately, valid proof of “clinical death”?), the code of practice has equated brainstem death with clinical death because of the fulfilment of two conjunctive clinical criteria:

[c]oncern is sometimes expressed over continuing function within the brainstem, occurring beneath the level at which any motor, somatosensory or breathing reflexes can be elicited and also over continuing function in other parts of the brain. However, as has already been indicated, both are irrelevant *when evaluating function against these clinical criteria of death* *resulting from irreversible cessation of brain*-*stem function, which demonstrate the permanent absence of consciousness and thus the ability to feel or do anything, along with the inevitable and rapid deterioration of integrated biological function* [emphasis added] (The Academy of Medical Royal Colleges 2008, p. 17).

Firstly, the code of practice assumes that the diagnosis of brainstem death is equivalent to clinical death because of an irreversible loss of the capacity for consciousness. Many clinicians in the UK have presented this assumption in medical literature as a confirmed fact when there is no supportive neuroscientific evidence (Cameron et al. 2016). As previously highlighted, Mr. Justice Hayden was told “that no patient has ever regained consciousness or awareness following brain stem death” (*Re: A (A Child)* 2015, para 11). But did absent brainstem function (reflexes) confirm the absence of capacity for regaining consciousness or awareness? Rapidly progressing neuroscience has improved the understanding of the neurophysiology of human consciousness and the clinical spectrum of disorders of consciousness (Zeman 2001). Functional neuroanatomy of human consciousness is far more complex than previously appreciated and depicted in the code of practice (Di Perri et al. 2014). Neuroscientific research has refuted the assumption that the locus of human consciousness lies exclusively in the brainstem (Rady and Verheijde 2016a). Functional neuroimaging has identified distinctive neural networks that mediate self-awareness and environmental awareness in the severely injured human brain (Vanhaudenhuyse et al. 2011; Di Perri et al. 2014; Gosseries et al. 2014). Additionally, as mentioned previously, 60 % of patients who are neurologically determined dead by absent function (reflexes) have a normal or minimally ischemic brainstem at autopsy (Wijdicks and Pfeifer 2008). Absence of brainstem reflexes does not necessarily equate with, nor confirm the absence of, capacity for consciousness or awareness (Peterson et al. 2014). Currently, there are no simple tests that can reliably verify the irreversible loss of capacity for consciousness or awareness in the severely injured human brain (Gosseries et al. 2014; Lutkenhoff et al. 2015). This is clearly illustrated by the “locked-in-syndrome” in which complete dependency on mechanical ventilation is necessary because of the loss of brainstem function, but the patient can still retain awareness of the surrounding environment and the self (Charland-Verville et al. 2015). The code of practice has not scientifically verified the neuroanatomical locus of consciousness, nor validated the tests of brainstem function as reliable indicators of irreversible loss of the capacity for consciousness or awareness. Secondly, the code of practice has indicated that “even if the body of the deceased [brainstem dead] remains on respiratory support, the *loss of integrated biological function* will inevitably lead to deterioration and organ necrosis within a short time” [emphasis added] (The Academy of Medical Royal Colleges 2008, p. 11). This claim was disproven almost two decades ago. Clinical data have demonstrated prolonged survival of brainstem dead patients with only mechanical ventilation and nutrition for as long as 14 years (Shewmon 1998a, b, 1999, 2001). Cumulative scientific evidence has refuted the assertion that brainstem death is associated with an irreversible disintegration of somatic biological functions or organ necrosis within a short time.

To summarize, the cessation of brainstem function (reflexes) in *Child A* did not imply irreversible loss of either capacity for consciousness or somatic integration of biological functions and failed to confirm the clinical criteria of death that are stipulated in the code of practice (The Academy of Medical Royal Colleges 2008, p. 17). We conclude by contending that the medical evidence presented was insufficient to apply the code of practice to determine brainstem death and to terminate life-support treatment at that particular time after acute hypoxic–ischemic brain injury in *Child A*. Also, had the code of practice been applied at the appropriate time after hypoxic–ischemic brain injury, the code itself would still have failed to verify the “clinical death” of Child A.

### Ethical and Societal Considerations

Good practice of medicine entails adherence to certain ethical principles. Child A’s case underscores the relevance of the “do no harm” principle in life-ending medical decisions in the ICU. The clinical uncertainty in neuro-prognosis and diagnosis should have been sufficient for a pause before proceeding with terminal withdrawal of life-support treatment. An avoidable loss of life can result if the medical decision is prematurely made to withdraw therapeutic interventions or beneficial treatment when there is a level of uncertainty that goes beyond what is generally understood to be clinically acceptable. It is difficult to prognose the neurological outcome or the future quality of a child’s life early after hypoxic–ischemic brain injury. Several studies (Gold et al. 2014; Ponz et al. 2016) have cautioned against an early withdrawal of life-support because of serious errors in prediction of a neurological prognosis after acute hypoxic–ischemic brain injury.

Some clinicians have nevertheless imposed their personal normative values on others based on their normative stance that survival with severe neurological disabilities is a worse outcome than death and that the continuation of life-support treatment is tantamount to “torture”, “inhumane”, “punishment”, or “degrading treatment” (Brierley et al. 2013). However, if life-support treatment is truly universally harmful and causative of unmanageable pain and suffering, then the critical care medicine practice and pediatric ICUs would not have grown globally over the past decades. On the contrary, premature discontinuation of therapeutically effective intervention or beneficial treatment is likely to cause distress and even death. The benefit of continuing life and avoiding premature death can outweigh the burden of treatment. Withdrawing mechanical ventilator support can result in acute asphyxia and distress which is generally difficult to detect and palliate in severe brain injuries (Rady and Verheijde 2016b).

Good medical care is multidimensional and requires attention to physical aspects, as well as emotional, spiritual, and social aspects of illness. These are essential components in patient-centered and family-centered medical care. Cultural competence and sensitivity of the medical staff are also the hallmarks of providing compassionate care to patients and families, especially in the presence of a potentially catastrophic and fatal illness (Larcher et al. 2015). The divergence of moral values and beliefs, as well as the imbalance in authority and power between healthcare teams and families, often result in conflict and adversarial confrontation:

A family’s concept of their child’s best interests is likely to be determined by their own system of values. A number of influences shape a family’s collective value systems; these include religious beliefs, political and cultural attitudes and life experiences. Parental values may not coincide with those of professionals. Disagreements may be aggravated by the power imbalance inherent in the healthcare professional/patient, child/parent relationship (Larcher et al. 2015, p. S11).

Therefore, the Royal College of Pediatrics and Child Health has cautioned against disregarding cultural diversity when making clinical decisions about forgoing treatment in children:

The UK Census… has confirmed that 1 in 10 children are classed as from minority ethnic groups and therefore decisions on limitation of treatment need to be underpinned by an understanding of cultural ‘diversity’. This remains a relatively under-researched area, but is important in the face of increasing cultural diversity in the UK (Larcher et al. 2015, p. S21).

Patients and families are vulnerable for several reasons. Firstly, they do not realize there is a wide variability among ICU clinicians in the decision-making about forgoing life-support treatment at the end of life (Sprung et al. 2015). Secondly, patients and families have limited or no ability to choose ICU clinicians who share or respect their values, which would mitigate the risk of variability in end-of-life decisions making (Zivot 2012). Thirdly, families are not generally informed about how death is defined and according to which criteria it is determined in British ICUs. The code of practice considers the absence of consciousness as the defining feature of death: “irreversible loss of those *essential characteristics* which are necessary to the existence of a *living human person* and, thus, the definition of death should be regarded as the *irreversible loss of the capacity for consciousness*” [emphasis added] (The Academy of Medical Royal Colleges 2008, p. 11). Fourthly, families are not informed that the presence or absence of consciousness cannot be reliably verified by current brainstem tests. Finally, clinicians who incorrectly claim that neurological (brainstem) death perfectly equates to biological death can cause families to distrust the healthcare team.

In the US, the President’s Council on Bioethics (2008) refuted the equivalency of neurological (whole brain) death with biological death in the “Controversies in the Determination of Death: A White Paper by the President’s Council on Bioethics”. Somatic integration and most of the bodily biological functions that are characteristic of living humans are preserved in neurological death, but not in biological death (The President’s Council on Bioethics 2008, p. 56). To salvage the equivalency of neurological death with human death, a philosophical definition of death was proposed. The determination of life or death in a whole organism, the President’s Council argued, is dependent upon the organism’s ability to perform fundamental vital work, i.e., to commerce with the surrounding world:

Determining whether an organism remains a whole depends on recognizing the persistence or cessation of the fundamental vital work of a living organism—the work of self-preservation, achieved through the organism’s need driven commerce with the surrounding world (The President’s Council on Bioethics 2008, p. 60).

In the context of death determination, the President’s Council limited the vital work of a living whole organism only to the capacity for consciousness and spontaneous breathing. If the whole organism has irreversibly lost the capacity for consciousness and spontaneous breathing, it cannot commerce with the surrounding world and should, therefore, be considered dead regardless of preservation of somatic integration. This philosophical redefinition of life and death has been criticized because of inherent biological and logical inconsistencies that result in erroneous (false positive) death determination in clinical practice (Shewmon 2009).

Meanwhile, the UK code of practice for neurological (brainstem) death determination has always required only cessation of the functions of the brainstem instead of the entire or whole brain (which includes the higher brain structures as well as the brainstem) (The Academy of Medical Royal Colleges 2008). We have argued that the tests of brainstem function, even if properly undertaken, cannot reliably confirm the two conjunctive clinical criteria of death set forth in the code of practice (loss of capacity for consciousness and loss of somatic integration of bodily biological functions). The President’s Council on Bioethics also emphasized the potential dangerous consequences of false positive or faulty death determination in only:

accepting ‘death of the brainstem’ rather than total brain failure, as a sufficient criterion for declaring a patient dead. Such a reduction, *in addition to being conceptually suspect, is* *clinically dangerous* because it suggests that the confirmatory tests that go beyond the bedside checks for apnea and brainstem reflexes are simply superfluous [emphasis added] (The President’s Council on Bioethics 2008, p. 66).

Pallis, a British neurologist and the founder of the brainstem death concept in the code of practice, believed that the capacity for consciousness and spontaneous breathing are a “sociological context for basic concepts of life and death… and … two definitive features of the human soul” (The President’s Council on Bioethics 2008, p. 65). Pallis interpreted that the brainstem was the locus of human soul and that in brainstem death the human soul departed from the physical body: “[t]he formulation is, in my opinion, merely a secularized restatement (in the language of modern neurophysiology) of such age-old notions as the ‘departure of the (conscious) soul from the body’ and the ‘loss of the breath of life’” (Pallis 1995, p. 20). In response to his critics, Pallis stated “[r]est assured moreover that the concept of brainstem death has a solid philosophical basis: one that would certainly be consonant with your notion of ‘loss of personal identity’” (Pallis 1995, p. 22). The code of practice has implicitly invoked a philosophical definition of death and, with that, a presumed sequence in determining death: loss of capacity for consciousness (and spontaneous breathing) = neurological death = departure of the ‘soul’ and/or loss of personal identity = human death. However, by unilaterally invoking a value-laden construct of death, the neurological criteria of death determination have been adopted without any national dialogue or public involvement. The code of practice has wrongly authorized a secular construct of death based on: (1) a mischaracterized broad societal acceptance of consciousness and spontaneous breathing as the dividing line between life and death, (2) a misaligned Pallis’ interpretation of the locus of the human ‘soul’ and the time of its departure from the body, and, (3) an unsubstantiated claim of the subsequent loss of personal identity. Consequently, the moral obligation of a pluralistic society to honor and respect diverse religious convictions to the greatest extent possible is being violated.

When the medical construct of brainstem death was endorsed in *Child A*, just as it was in previous British cases, the court either inadvertently or consciously chose to allow one value-based definition of death (i.e., a secular viewpoint which focuses on a philosophical definition of loss of personhood) to dominate over another equally-respected and respectable value-based system (i.e., a religious viewpoint which focuses on biological definition and sanctity of life). This has the potential to deprive residents of their constitutional rights and transgress diverse cultural and religious value systems in a pluralistic society (Biggar 2015). Child A’s case raises the question of whether it is appropriate that a decision which holds such monumental societal implications should be decided retrospectively by the judicial process rather than prospectively through the legislative process. It is worth mentioning that the courts have repeatedly refused to rule on whether the law should allow physician-assisted suicide, preferring for Parliament to undertake this task instead (*R (on the application of Nicklinson and another) v Ministry of Justice* 2014)). It is surprising that the judicial system has not embraced a similarly guarded position on rulings about legal death determination despite the far more serious repercussions involved. After all, the legal determination of death assumes an extremely important social boundary (Magnus et al. 2014, p. 894). The status of dead or alive signifies whether an individual should still be recognized as a person with constitutional rights; when his last will and testament become effective and his estate will devolve; and when his remains can be buried or cremated (Magnus et al. 2014; Shaw 2014). More importantly in the clinical context, it signifies the point beyond which healthcare professionals are no longer under a duty to provide medical treatment to the patient, and indicates when life-support treatment can be discontinued.

The construct of brainstem death also enables the procurement of transplantable vital organs from heart-beating donors before biological death (Shah 2014, p. 106; Larcher et al. 2015; Rady and Verheijde 2016c). A brainstem dead child is a potential donor of vital organs, and mechanical ventilation and medical treatment would be continued until the removal of organs:

I [Mr. Justice Hayden] cannot conceive of any circumstances in which the Coroner should seek to intervene, where a body remains ventilated, beyond those circumstances concerning the removal of organs where the family are consenting (*Re: A (A Child)* 2015, para 22).

Although organ donation was not a consideration in *Child A*, Brierley and Larcher have called for major legal, ethical, and cultural changes in the UK society to overcome the end-of-life barriers to pediatric organ donation: “[u]nlike the UK, the USA, Australia, Canada and many European countries *accept the concept of brain death in infants* and concomitant certification that *can lead to DBD* [donation after brain death]” [emphasis added] (Brierley and Larcher 2011, p. 1176). Brierley has also advocated for elective initiation and continuation of mechanical ventilation in eligible neonates and infants to maximize organ donation and transplantation in the UK (Jivraj et al. 2016).

If the legal system is supportive of a medical definition of death with a specific dominant philosophical underpinning intended to serve the interests of specific subgroups of clinicians, this should raise several concerns. Is the judiciary assuming joint legislative roles with the medical profession in favoring a death determination which facilitates pediatric organ donation? If so, should there not be more transparency by clarifying this point in the judgment so that the public are sufficiently informed of the ramifications of the judicial standpoint? Otherwise, not only is organ procurement operating in an opaque manner, the recognition of only a single definition of death also undermines respect for religious freedom. This is enshrined in Article 9 of the European Convention on Human Rights, “[e]veryone has the right to freedom of thought, conscience and religion; this right includes the freedom to…manifest his religion or belief, in…practice and observance” (European Convention on Human Rights 1950). The latter is particularly marked when Mr. Justice Hayden refused to accommodate Child A’s parents’ religious viewpoint that brainstem death does not equate to death in Islam. The High Court’s decision sends a reverberating message that the definition of death proffered by the medical profession applies without exception to faith communities. So resolute and uncompromising was this stance that Mr. Justice Hayden refused, as highlighted, to accommodate even the parents’ alternative request to continue mechanical ventilation in Child A until arrangements were made for air medical transportation to Saudi Arabia. This informs us that the acceptance of the medical definition of death is so total and unreserved that no allowance is made even for citizens of other countries who have a different concept of when death occurs. This decision also implies that British Muslims or Jews, for example, who object to brainstem death determination will not be allowed continuation of mechanical ventilation so that arrangements can be made for transferring to an alternative medical facility or to another country like Saudi Arabia or Israel.

## A Comparative Perspective

The judicial ruling in *Child A* contrasts sharply with the US approach in the case of Jahi McMath (*Winkfield v. Childrens Hospital Oakland* (2013)). Jahi, who was 13 years old when she underwent a surgical procedure in 2013 to correct sleep apnea, was declared brain dead when post-operative complications resulted in hypoxic–ischemic brain injury. When doctors at the Oakland Children’s Hospital in California attempted to withdraw mechanical ventilation, the family similarly objected to this decision based on religious grounds (Johnson 2016). Jahi’s parents argued that from their Christian faith perspective, death occurs when the heart stops beating and that Jahi was still alive as her heart was still beating spontaneously. The McMath family petitioned the court to prevent the hospital from withdrawing life-sustaining treatment. Ruling in favor of Jahi’s doctors, Judge Evelio Grillo pronounced her dead under California Law. This was in accordance with California’s Uniform Determination of Death Act (UDDA) which states that “[a]n individual who has sustained either (1) irreversible cessation of circulatory and respiratory functions, or (2) irreversible cessation of all function of the entire brain, including the brain stem, is dead” (Uniform Determination of Death Act 1981). What was particularly noteworthy about the judgement, however, was how the judge allowed Jahi’s ventilated “dead body” to be handed over to the coroner through an elaborate legal charade, to mark that she was indeed dead in the eyes of California law. The coroner then issued a death certificate and handed Jahi, still on mechanical ventilation, back to her family. This was not for the usual purpose of burial or cremation, but so that she could then be transported to an undisclosed medical facility in New Jersey which was willing to continue life-sustaining treatment. Jahi, in the process, was treated not as a patient nor a dead body since “living patients are not sent to the morgue, and dead bodies are not actively ventilated” (Johnson 2014, p. 36). The judgement was consistent with Section 1254.4 of the California Health and Safety Code which instructs that if:

the patient’s legally recognized health care decision-maker, family or next of kin voices any special religious or cultural practices and concerns of the patient or the patient’s family surrounding the issue of death by reason of irreversible cessation of all functions of the entire brain of the patient, the hospital *shall make reasonable efforts to accommodate those religious and cultural practices and concerns* [emphasis added] (Nguyen 2015, p. 408).

It is noteworthy that although the McMath family’s exercise of religious beliefs was burdened by the absence of a religious exception in California’s UDDA, the request for transferring Jahi to an alternative medical facility was facilitated (Luce 2015, p. 1149). Jahi was transferred to a medical facility in New Jersey where the law allows patients and families to reject brain-based determination of death. In fact, the law proscribed a declaration of death being made on the basis of neurological criteria where “such a declaration would violate the personal religious beliefs of the individual” (New Jersey Declaration of Death Act 1991). In such a circumstance, death is to be declared only on the basis of cardio-respiratory criteria. Although the US has not yet gone so far as to have a religious exception recognized in all states (Delaney 2010), the fact that it is recognized in New Jersey allowed the court to exercise some degree of flexibility when it facilitated the transport of Jahi on mechanical ventilation to another medical facility (Lewis et al. 2016).

It is curious that Mr. Justice Hayden made no reference to the McMath case in the High Court’s judgement despite the publicity of this case, nor did he give serious consideration to a similar request to relocate Child A while still ventilated to Saudi Arabia where the parents claimed that mechanical ventilation would never be withdrawn in similar circumstances. This raises another question: Why was this conclusion made with such inexplicable urgency, when it was logistically possible to repatriate a ventilated Child A to Saudi Arabia? New advances in medical technology and critical care delivery have enabled safe air medical transportation of critically ill patients on mechanical ventilation across continents. This was safely accomplished when Jahi McMath was transported across North America from the western state of California to the eastern state of New Jersey. Was the decision in *Child A* made solely because of a desire not to be inconsistent with the established legal position? If so, the High Court could have at least engaged in the same legal creativity as in the McMath case. Judge Grillo’s decision, far from compromising Californians’ faith in the court system, demonstrates flexibility and compassion when dealing with religious objections to brain death. Instead, as observed by Brierley (2015), a pediatric intensivist at London’s Great Ormond Street Children Hospital, “religious control over how death can be verified does not seem to be lawful in the UK.” It appears that the secularist viewpoint in the determination of death is set to prevail over religious values in the UK. This dominance of the secularist viewpoint in the UK can be sharply contrasted with the survey findings that most clinicians in the US would accommodate and respect religious objection of families to death determination by neurological criteria (Ayeh et al. 2016).

## Recommendations

The case of *Re A (A Child)* came to the attention of the British judiciary approximately four decades after the medical fraternity in the UK adopted brainstem death as the determinant of death. Although this definition has still not been put on statutory footing, the courts have consistently endorsed the code of practice issued by the Academy of Medical Royal Colleges for the determination of neurological death. The judgment in the present case highlights not only the extent to which the judiciary continues to adopt a passive and unquestioning acceptance of the code, but that it is recognized as the sole determinant of death in British ICUs. As discussed, these suggest that the High Court was both unconcerned with whether the code was applied correctly in this case and oblivious to the growing scientific literature which cast doubt on the reliability of the mechanisms outlined in the code. More fundamentally, the refusal to consider religious conceptions of death meant that room is not created for religious dissension from the secular code. In light of these concerns, several recommendations are outlined below.

We urge against making an irreversible decision about early withdrawal of life-support treatment after acute hypoxic–ischemic brain injury. Instead, treatment should be maintained until greater clarity of neuro-prognosis is possible. Scientifically validated tests should be utilized in the determination of neuro-prognosis and diagnosis since current tests of brainstem function (reflexes) and static neuroimaging studies cannot reliably predict the irreversible loss of capacity for consciousness or neurological recovery. We also recommend that medical staff should be educated and trained in cultural sensitivity and communication to reduce the frequency of conflicts with patients and families with diverse cultural values. In end-of-life conflicts involving terminal withdrawal of life-support treatment, patients and families are vulnerable because they have no choice in selecting clinicians who share or respect their values. Society should protect these vulnerable individuals and provide them with a fair and due process in the resolution of intractable conflicts.

We further recommend that the British judiciary and/or legislators accommodate and respect residents' religious rights and commitments when secular conceptions of death based on medical codes and practices conflict with a traditional concept well-grounded in religious and cultural values and practices. Respecting cultural values and religious beliefs strengthens the protection of human rights in a multicultural society.

Neurologically-based (brainstem) death determination invokes a soul-less secular death definition which should not overrule traditional death definition upholding religious values and sanctity of life. Secular death definition can support organ donation and transplantation, but is conditional upon societal trust in the scientific authenticity of death determination (Rady and Verheijde 2016d). As public distrust may undermine support for cadaveric donation (Martin et al. 2015), this underlines the need for public debate and room for the adoption of more than one definition of death in a multi-religious society like the UK (Choong 2013).

## Conclusions

The determination of neurological (brainstem) death does not fulfill the two clinical criteria of death that are stipulated by the Academy of Medical Royal Colleges in the code of practice: (1) irreversible loss of capacity for consciousness, and (2) loss of somatic integration of biological bodily functions. Scientific advances have refuted the assertion that tests of brainstem function can provide conclusive evidence in the confirmation of clinical death. The construct of “brainstem death” is philosophically grounded in the loss of personhood which is a secular definition of death with vested interests in advancing the organ donation and transplantation practice. The legal system should not favor a secular definition of death that transgresses deeply ingrained religious values about the sanctity of life and deprives residents of their right to fully observe their religious teachings. We urge the legal system to adopt a more compassionate approach to death determination that is respectful of cultural and religious belief systems in a pluralistic society like the UK.

## Electronic supplementary material

Below is the link to the electronic supplementary material.

Supplementary material 1 (RTF 161 kb)
