# Supplementary material for: Re A (A Child) and the United Kingdom Code of Practice for the Diagnosis and Confirmation of Death: Should a Secular Construct of Death Override Religious Values in a Pluralistic Society?
Source: HEC Forum. 2016 Aug 4;30(1):71–89. doi: 10.1007/s10730-016-9307-y (PMC5847223; doi:10.1007/s10730-016-9307-y)
Supplement: Supplementary file 1 — Supplementary material 1 (RTF 161 kb) [file 10730_2016_9307_MOESM1_ESM.rtf]

Apple Transcription Limited		1-956-3369-19/ma
0845 604 5642 		v.4
Neutral Citation Number: [2015] EWHC 443 (Fam)

IN THE HIGH COURT OF JUSTICE
FAMILY DIVISION
MANCHESTER DISTRICT REGISTRY
Civil Justice Centre
1 Bridge Street West
Manchester
M60 9DJ

Date: Thursday, 12th February 2015


Before:

THE HONOURABLE MR JUSTICE HAYDEN


In the matter of:

Re: A (A Child)


______________________

Counsel for the Applicant NHS Trust:	MISS CAVANAGH

The First Respondent Father, Mr A, appeared In Person

The Second Respondent Mother did not appear and was not represented

Counsel for the Third Respondent the Senior Coroner for Manchester:	MR BURROWS
______________________


APPROVED JUDGEMNT


Transcribed from the Official Tape Recording by
Apple Transcription Limited
Suite 204, Kingfisher Business Centre, Burnley Road, Rawtenstall, Lancashire BB4 8ES
DX: 26258 Rawtenstall – Telephone: 0845 604 5642 – Fax: 01706 870838

Number of Folios: 51
Number of Words: 3,648

DRAFT JUDGMENT

1.	THE	JUDGE:  In this application, the Central Manchester NHS Foundation Trust, represented by Miss Cavanagh, seek declarations in relation to a child, Child A, who was declared clinically dead at 10.10am on 10th February 2015.  That declaration was confirmed by two brain stem tests, the latter taking place at 17.30 hours the same day, confirming the results of the earlier test and, therefore, supporting the declaration i.e. that brain stem death had occurred at 10.10am on that date.
2.	Child A is a 19-month-old boy.  His parents are Saudi nationals and they have been living in the United Kingdom for the last two years.  The father, who has been present in court throughout this application, is studying for a PhD.  Child A has a brother, Child B, who is nearly 6 years of age and a sister, Child C, who is 2½.
3.	Tragedy struck this family.  On 6th February 2015, Child A choked on what appears to have been a tiny piece of fruit.  Shortly before 2.30 in the afternoon that day, his mother found him choking and it seemed he quickly became unconscious.  The mother is not at court.  She is with her other children and, understandably, she is profoundly distressed.  That afternoon, she telephoned the ambulance immediately.  It arrived promptly and the paramedics were immediately able to perform bag valve mask ventilation en route to the hospital.  
4.	By the time Child A arrived at the hospital, he had unrecordable saturations.  His position was grave.  It was plain to everybody that Child A was in severe respiratory distress on his arrival.  Though he was breathing spontaneously, he had a marked airway obstruction.  Tracheal intubation and cricothyroidotomy did not relieve the situation.  Hypoxia and ischemia led to cardiac arrest and, at 15.11 hours, that is to say easily within half an hour of arriving at the hospital, CPR was commenced.  
5.	In addition to intubation and needle insertion into the trachea, performed by the consultant anaesthetist, Child A saw an ENT consultant who was able to operate immediately to remove the foreign body, via endoscopy and to retrieve a small stalk which appeared to have come from a satsuma.  By 15.31 hours, cardiac output had been re-established and Child A was transferred to paediatric intensive care.  There he remained fully ventilated and assisted by a neuro-protective regime which involves the application of a group of therapies which, to the best understanding of medical science, is thought to reduce the extent of brain swelling and the raising of intracranial pressure, following hypoxic ischemic insult.  So Child A was heavily sedated, the CO2 control was carefully balanced and he was rested at an angle of 30 degrees, thought to offer the best neuro-protection to reduce swelling but it was entirely obvious to all the nurses and staff that the outlook for Child A was profoundly bleak.
6.	Recognising that, Dr Stephen Playfor, a consultant paediatric intensivist with over 13 years' experience, told me that he considered it wise to move directly to MRI scanning and such was undertaken on 7th February. The scan, perhaps not unpredictably, revealed extensive severe ischemic changes involving the grey matter of Child A's brain.  It could not have been more obvious, both from the clinical presentation of this little boy and of the content of the MRI scan that the brain injury was so extensive that it was something from which it was impossible for Child A to survive.
7.	It must have been almost impossible for his mother and father to understand how a day which began so ordinarily on 6th February could have deteriorated so rapidly and unexpectedly into what the father has described to me as “a living hell”.  In my view, it is almost impossible for any adult to absorb the extent of the parent's distress.  Human instinct senses that level of pain and recoils from it.  Ultimately, all we, the lawyers and doctors, can do is to offer Mr and Mrs A and their family our profound sympathies and condolence.  This is a Muslim family.  Mr A has wanted to cleave to what thread of life he perceives his son still to have.  He has listened to the evidence of Dr Stephen Playfor and his response is, in my assessment, one of bewilderment, frustration and anger perhaps an inevitable component of such unimaginable grief.
8.	The brain stem tests to which I have referred were undertaken on 10th February 2015.  By this stage, Child A had begun to show signs of significant cardiovascular instability.  He had developed diabetes insipidus.  This occurred in consequence of impaired hormone production from his profoundly injured brain.  At hospital, he was given an infusion of vasopressin to stabilise the diabetes, as I understand it, in order to protect his kidneys and to seek to fend off dehydration.
9.	The brain stem tests followed well established clinical guidelines.  Despite the impressive name, they are, in fact, simple bedside tests: an assessment of the pupils' reaction to light; corneal reflexes; cough reflex; evidence of any respiratory movement; apnoea tests; and gag reflex.  For each test, on those two separate occasions, the results pointed overwhelmingly and sadly, conclusively, to brain stem death.
10.	One of the questions posed by the father here was whether brain stem death is synonymous with clinical/legal death.  The father certainly does not consider the two to be equivalent. This seems to me at least as rooted in his Muslim beliefs as in his basic paternal instinct.  I have considered the Code of Practice devised by the Academy of Medical Royal Colleges (2008) both in relation to “The Diagnosis and Confirmation of Death” following irreversible cessation of brain stem function and in relation to the conclusions and recommendations of the 1991 “Report of a working party of the British Paediatric Association on the diagnosis of brain-stem death in infants and children”.  
11.	Dr Playfor, who I found to be an impressive, kind and reflective witness, gave me this assistance: he told me brain stem death, does not equate to the death of the whole brain.  There are studies that demonstrate that you can have electrical activity in some areas of the brain after brain stem death is established.  The key point, he said, is that no patient has ever regained consciousness or awareness following brain stem death.  Dr Playfor went on to explain the reason for that in language which I found to be simple and accessible.  The nerves which generate the breathing mechanism and maintain the integrity of the heart rate are all connected to the brain stem.  In simple terms, when the brain stem dies, it is impossible for a patient to breathe unassisted.
12.	The Code of Practice, which applies to infants, children and adults who are comatose and being ventilated, define death as:
“2.1 Death following the irreversible cessation of brain-stem function 

The irreversible cessation of brain-stem function whether induced by intra-cranial events or the result of extra-cranial phenomena, such as hypoxia, will produce this clinical state and therefore irreversible cessation of the integrative function of the brain-stem equates with the death of the individual and allows the medical practitioner to diagnose death. 

Three things should be noted in this regard: 
First, the irreversible loss of the capacity for consciousness does not by itself entail individual death. Patients in the vegetative state (VS) have also lost this capacity (see section 6.9). The difference between them and patients who are declared dead by virtue of irreversible cessation of brain-stem function is that the latter cannot continue to breathe unaided without respiratory support, along with other life-sustaining biological interventions. This also means that even if the body of the deceased remains on respiratory support, the loss of integrated biological function will inevitably lead to deterioration and organ necrosis within a short time. 

Second, the diagnosis of death because of cessation of brain-stem function does not entail the cessation of all neurological activity in the brain. What does follow from such a diagnosis is that none of these potential activities indicates any form of consciousness associated with human life, particularly the ability to feel, to be aware of, or to do, anything. Where such residual activity exists, it will not do so for long due to the rapid breakdown of other bodily functions. 
In short, while there are some ways in which parts of the body may continue to show signs of biological activity after a diagnosis of irreversible cessation of brain-stem function, these have no moral relevance to the declaration of death for the purpose of the immediate withdrawal of all forms of supportive therapy. It is for this reason that patients with such activity can no longer benefit from supportive treatment and legal certification of their death is appropriate.

The current position in law is that there is no statutory definition of death in the United Kingdom. Subsequent to the proposal of the 'brain death criteria' by the Conference of Medical Royal Colleges in 1976,3,4 the courts in England and Northern Ireland have adopted these criteria as part of the law for the diagnosis of death.5,6 There is no reason to believe that courts in other parts of the United Kingdom would not follow this approach.

Third, there may also be some residual reflex movement of the limbs after such a diagnosis. However, as this movement is independent of the brain and is controlled through the spinal cord, it is neither indicative of the ability to feel, be aware of, or to respond to, any stimulus, nor to sustain respiration or allow other bodily functions to continue.”

13.	The irreversible loss of the capacity for consciousness does not by itself establish an individual's death.  The difference between patients in vegetative state and patients who are declared dead by virtue of irreversible cessation of brain stem function is that the latter cannot continue to breathe unaided without respiratory support and other life sustaining interventions. The Code of Practice provides:
“CHAPTER 6: THE DIAGNOSIS OF DEATH FOLLOWING IRREVERSIBLE CESSATION OF BRAIN-STEM FUNCTION
Concern is sometimes expressed over continuing function within the brain-stem, occurring beneath the level at which any motor, somatosensory or breathing reflexes can be elicited and also over continuing function in other parts of the brain. However, as has already been indicated, both are irrelevant when evaluating function against these clinical criteria of death resulting from irreversible cessation of brain-stem function, which demonstrate the permanent absence of consciousness and thus the ability to feel or do anything, along with the inevitable and rapid deterioration of integrated biological function.”

14.	That which Lord Browne-Wilkinson in Airedale NHS Trust v Bland [1993] A.C. 789 viewed with apparent incredulity, now 20 years ago, is common place in contemporary medical practice i.e. assisted ventilation post brain stem death. His words repay revisiting:
“This has led the medical profession to redefine death in terms of brain stem death, i.e., the death of that part of the brain without which the body cannot function at all without assistance. In some cases it is now apparently possible, with the use of the ventilator, to sustain a beating heart even though the brain stem, and therefore in medical terms the patient, is dead; "the ventilated corpse.” [Page 878F]

15.	Applying all this, to Child A's tragic circumstances, I conclude that by 10.10 am on 10 February 2015 the criteria for death had been established.  
16.	In the meantime, Child A has remained fully ventilated in 35 percent oxygen.  He makes no respiratory effort.  He is unable to do so.  His temperature is maintained on an external cooling blanket and, as I understand it, he continues to receive infusion of vasopressin to control his urine output and balance his fluids.  Enteral feeds are not being tolerated and, insofar as it is clinically relevant, laboratory results are, essentially, normal, apart from fluctuations in his serum, sodium, urea and creatinine levels, which are, of course, linked to his diabetes.
17.	Child A's parents have simply been unable to contemplate turning off ventilatory support.  Mr A clings on to any sign that may undermine these catastrophic medical conclusions, pointing to the twitching and retraction of Child A's legs, which are spinal, not cerebral reactions.  That seems to me to be entirely understandable.  He and his wife, whose lives have been rocked in the course of the last week, have tried to put together a package of measures to take Child A (whilst continuing his ventilation) to Saudi Arabia.  Some of the papers indicate that the father favoured that course because he wished Child A to die there but I was not at all surprised to hear from him, in evidence, that his real motivation was to take Child A to Saudi Arabia in the hope that he might live.  In Saudi Arabia, the father told me, for religious reasons, a life support machine would never be switched off. This would appear to be correct. It is perhaps also important to note that in the USA the parallel guidance identifies 'whole brain death' as the legal definition of death, specifically rejecting the UK Guidelines as too narrow (“Controversies in the determination of death”: A White Paper by the President's Council on Bioethics, Washington, DC. page 65-66).
18.	Dr Playfor to my mind had tried to hold the balance between his sympathy for these grieving and highly distressed parents and his duty to his patient.  He was prepared to, and did, contact the medical liaison officer at the Saudi Arabian Embassy, exploring, perhaps somewhat improbably on reflection, the possibility of taking Child A in his present condition to Saudi Arabia.  
19.	Whilst he and the family were grappling with this awful dilemma, the Coroner had become acutely conscious that this little boy had now been brain stem dead, and therefore dead, for approaching 48 hours. In accordance with protocol the Coroner had been informed.  He wrote to Professor Pearson, the Trust's clinical director, in these terms:
“Technically, I have assumed jurisdiction over the body.  It seems wholly inappropriate for a deceased body to be intubated and ventilated when this is futile and, to my mind, unethical.  Accordingly, I must ask you to cease this and extubate him so that his body can be moved to the mortuary from which it can be released to his parents.  If the family wish to repatriate his body to Saudi Arabia, then I will provide an out of England certificate.  Obviously, your clinicians will need to communicate this to the parents and allow a short but reasonable time for the parents to be with him pending the extubation.”

20.	Mr Nigel Meadows, the Senior Coroner for Manchester, might well reflect now whether that kind of language was suitable in such sensitive circumstances.  Properly, to my mind, the Trust took a different course, bringing the matter before the High Court and inviting this court to resolve the issues identified in the draft declarations.  
21.	In his measured submissions, Mr Burrows acknowledged that both the Coroner and the High Court (under its parens patriae and/or inherent jurisdictional powers) had jurisdiction over the body. No Coroner appears to have asserted such authority in circumstances where life support remains engaged, following brain stem death.  I have been referred by counsel to “Jervis on Coroners”(13th Edition), the standard textbook.  At paragraph 5-14 (page 81) it states as follows:
“The coroner may also be faced with the difficult task of deciding whether a body in his area is actually dead, for instance when it is connected to a life support machine in an irreversible coma...  it appears that once a person has suffered brain stem death which no medical treatment is able to reverse, the person is 'dead' for the purposes of the coroner acquiring jurisdiction even whilst a machine ventilates the body.”  

That proposition is said to be supported by Mail Newspapers v Express Newspapers [1987] FSR 90; Airedale NHS Trust v Bland [1993] AC 789.  The footnote also refers to Thurston's Coronership: 3rd Edition 1985, which sets out the view that I have just recorded but also the opposing one, that while the heart beats and the blood circulates, there is no “dead” body i.e. for the purposes of establishing the Coroner's jurisdiction. I note that the distinguished authors also make the following observation which, in tone, seems to imply that they regard it as self evident: 
“Of course, in practice no Coroner would insist on taking possession of the body were it was still connected to a life support system.”

22.	I associate myself entirely with those observations. I cannot conceive of any circumstances in which the Coroner should seek to intervene, where a body remains ventilated, beyond those circumstances concerning the removal of organs where the family are consenting. Any other approach I regard as likely to generate immense distress and contribute to an atmosphere where sound judgment may be jeopardised. 
23.	Section 15 Coroners Act 2009 grants a Senior Coroner jurisdictional powers to remove a body:


“15	Power to remove body

E+W
This sectionnoteType=Explanatory Notes has no associated 
(1) A senior coroner who—
(a) is responsible for conducting an investigation under this Part into a person's death, or
(b) needs to request a post-mortem examination under section 14 in order to decide whether the death is one into which the coroner has a duty under section 1(1) to conduct an investigation,
may order the body to be removed to any suitable place. 
(2) That place may be within the coroner's area or elsewhere.
(3) The senior coroner may not order the removal of a body under this section to a place provided by a person who has not consented to its being removed there.
This does not apply to a place within the coroner's area that is provided by a district council, a county council, a county borough council, a London borough council or the Common Council.” 

24.	As the manner in which these provisions are drafted indicates, they are designed to facilitate the investigation of the cause of death.  The facts of this case are a reminder once again that in a multi-cultural society there has to be recognition that people, particularly those with strong religious beliefs, may differ with medical professionals as to when death occurs. In the Christian, Muslim and Jewish faiths the concept of the 'breath of life' has ancient and important resonance. It is hardly difficult to understand why the still breathing body is regarded as alive, even though 'breath' may be entirely delivered by machine. An insistence on a legally precise definition of death to trigger the involvement of the Coroner, in such challenging circumstances is, in my judgment, so obviously wrong as to be redundant of any contrary argument.
25.	For the avoidance of doubt all the advocates here have agreed that the High Court Family Division has the relevant jurisdiction. This clearly exists both under the parens patriae (which has its very origins in the responsibility, for 'a ward of court' and the body of the ward) and pursuant to an application for declaratory relief made under the inherent jurisdictional power.  The interrelationship between the two is sometimes subtle but, either separately or together, they authorise this court to make declarations in respect of what I must now regard as Child A's body. 
26.	Whilst expressing profound respect for the father's views, the time has now come to permit the ventilator to be turned off and to allow Child A, who died on 10th February, dignity in death.  For those reasons, I propose to make the declarations sought by the Trust, with the indicated amendments, confident that this hospital will do everything they can to make this inevitably painful process as dignified as possible for all concerned.  I would only add my profound condolences to Mr and Mrs A and to Child B and Child C.  
27.	I am very clear that should a difference of view arise between treating clinicians and family members in circumstances where assisted ventilation is continuing, any dispute, if it cannot be resolved otherwise,  should be determined in the High Court, not under coronial powers. 
[Judgment Ends]
